# From Passive Drift to Olfactory Detection? A Late‐Stage Oceanic Juvenile Hawksbill's 9000‐km Journey Across the Western Indian Ocean

**DOI:** 10.1002/ece3.74475

**Published:** 2026-10-30

**Authors:** Antoine Laforge, Phillipe Gaspar, Jérôme Bourjea, Manon Nivière, Katia Ballorain

**Affiliations:** ^1^ Centre d'Étude et de découverte des Tortues Marines (CEDTM) Saint‐Leu La Réunion France; ^2^ Mercator Ocean International Toulouse France; ^3^ MARBEC University of Montpellier, CNRS, Ifremer, and IRD Sète France

**Keywords:** hawksbill turtle, ocean currents, oceanic‐neritic transition, olfactory orientation, satellite telemetry

## Abstract

The mechanisms underlying the transition of juvenile sea turtles from oceanic to neritic habitats remain poorly understood, as the oceanic stage is rarely observed directly. Here, we report a 201‐day satellite track of a juvenile hawksbill turtle ( *Eretmochelys imbricata* , curved carapace length of 38 cm) that traveled 9129 km from the Mozambique Channel to Huvadhu Atoll, Maldives. Current reanalysis data indicate that 74% of this distance resulted from passive transport by regional ocean currents. However, upon approaching within 50 km of the atoll, the turtle showed a marked, active course correction, swimming against weak currents while positioned downwind and downstream of the atoll, a low‐lying landform (~1 m above sea level) that would have been invisible by eye at such a distance. This pattern raises the possibility of olfactory detection of the atoll and provides, to our knowledge, a first indication of olfactory‐based island detection guiding habitat recruitment in a juvenile turtle, a mechanism previously documented only in breeding females. This track suggests a potential two‐scale process of oceanic‐to‐neritic habitat transition: a broadly stochastic oceanic phase shaped by passive dispersal, followed by fine‐scale, sensory‐guided settlement once the turtle nears a suitable habitat.

## Graphical abstract

A juvenile hawksbill turtle ( *Eretmochelys imbricata* , CCL = 38 cm) was tracked for 201 days across 9129 km from the Mozambique Channel to Huvadhu Atoll, Maldives, with ocean current reanalysis showing that 74% of this distance resulted from passive drift by regional surface currents. Upon nearing the atoll, however, the turtle actively swam while positioned downwind of the island, in a pattern consistent with olfactory detection of the target habitat. This provides, to our knowledge, the first direct evidence of olfactory‐guided island detection in a juvenile sea turtle, a mechanism previously documented only in adult breeding females.

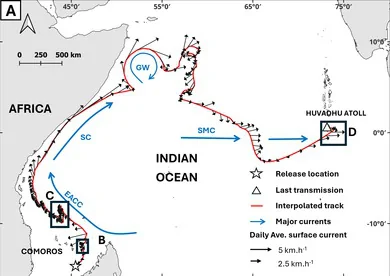

## 1 Introduction

Hawksbill turtles ( *Eretmochelys imbricata* ) are thought to transition from oceanic habitats, where they spend their first years, to nearshore coral reef environments once they reach a species‐ and population‐specific size threshold, estimated at 15.7–35.0 cm of curved carapace length (CCL; Bolten 1999) in the western Atlantic (Avens et al. 2021). The mechanisms and movement patterns associated with the end of the oceanic phase and governing the selection of neritic habitat remain poorly documented, as this ontogenetic habitat shift is rarely observed directly. Small juveniles are difficult to capture during their oceanic phase and, as a result, their habitat use during this period remains a major knowledge gap. Tracking juveniles further requires miniaturized satellite tags, which are constrained by limited battery life and often detach as turtles grow (Candela et al. 2024). Consequently, most tracks end before the transition from oceanic to neritic habitats can be observed.

## 2 Track Description and Methods

Here, we report the satellite track of a juvenile hawksbill turtle (CCL = 38 cm) that traveled from the northern Mozambique Channel (14°47′10.6″ S, 44°57′14.2″ E) to the Maldives (0°30′04.1″ N, 73°17′59.6″ E), corresponding to a straight‐line displacement of 3545 km from its release location. The juvenile, named *Naty*, was accidentally entangled on net below a Fish Aggregating Device and captured by a Spanish Purse Senner approximately 200 km south of Mayotte on 3 May 2010. The individual was released equipped with an Argos SPOT‐5 S244A Wildlife Tag (71 × 54 × 24 mm—length×width×height) and weighed 115 g; PTT ID 49583 as part of a collaborative program involving fishers (Ciccione and Bourjea 2010). The tag was attached to the second vertebral carapace scute with epoxy resin (Power‐Fast; Powers Fasteners Inc., Wieringerwerf, the Netherlands). The tag and attachment materials (< 150 g, for an estimated body mass of 5.5 kg; Hawkes et al. 2014) represented less than 3%, well below the recommended 5% threshold of body mass.

To characterize the oceanographic conditions encountered during this journey, we extracted surface current data from the GLORYS12 reanalysis (Lellouche et al. 2018) along the track. Following the methodology described in (Laforge et al. 2023), outlier positions were removed and the track was interpolated at 24‐h intervals using the R package *animotum* (Jonsen et al. 2023). GLORYS12 surface current vectors were then collocated with each interpolated turtle position. To investigate the hypothesis that the turtle may have detected nearby land through olfactory cues, we also extracted the 10 m zonal (u) and meridional (v) wind components from the ERA5 reanalysis. Wind fields and current fields were examined at locations and dates where the turtle passed within 100 km of land to determine whether the individual was positioned downwind or downstream of potential habitats and could therefore have been exposed to airborne or waterborne chemical cues originating from nearby islands or coastlines.

## 3 Results & Discussion

### 3.1 Trajectory Description

The tag transmitted for 201 days, until 28 November 2010, over which the turtle traveled a total distance of 9129 km. After release, the turtle initially moved northward through the Mozambique Channel, reaching the Banc du Geyser, a submerged rocky reef, generally 5–30 m deep, on 11 May, where it remained for 7 days exploring the eastern part of the bank. The turtle then resumed its northward movement (Figure 1B), passing successively 65 km west of Glorieuses on 20 May and 95 km west of Aldabra on 23 May. On 7 June, while located 350 km north of the Comoros archipelago and 200 km west of Aldabra, the turtle reversed its trajectory in the open ocean and began heading southward (Figure 1C), before shifting to a clear westward trajectory along the Horn of Africa. It then crossed eastward through the northwestern Indian Ocean, eventually reaching Huvadhu Atoll in the Maldives, 3545 km in a straight line from its release location, on 22 October 2010 (Figure 1D), where it settled. The individual subsequently remained in the southern part of the atoll for 37 days before transmissions ceased.

**FIGURE 1:**
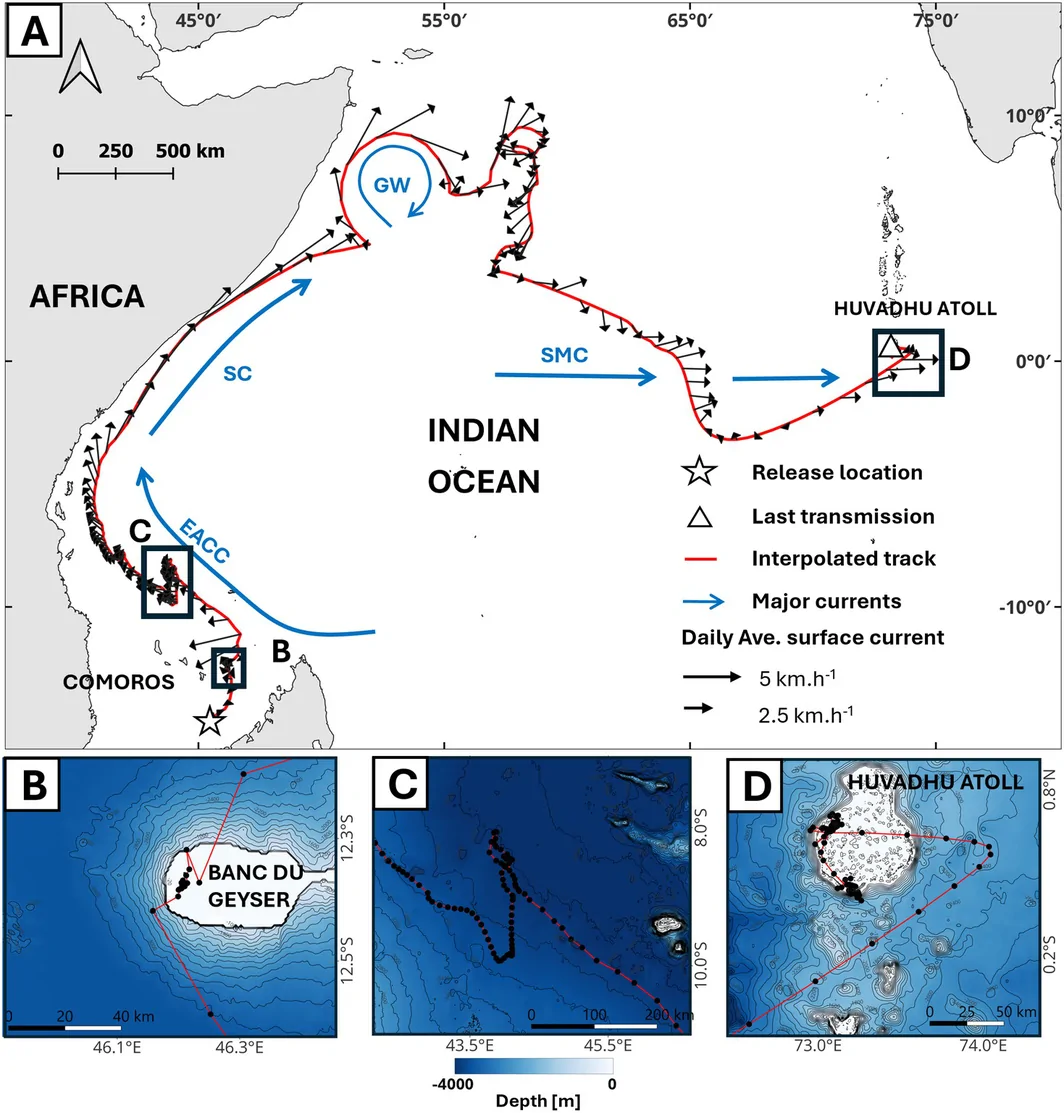
(A) Daily interpolated track of the juvenile hawksbill Naty (red line) from the release location in the Mozambique Channel (white star) to the final transmission site in the Maldives (white triangle), where the individual appears to have settled. Encountered drift was estimated from daily surface current vectors derived from the GLORYS12 reanalysis and collocated with each interpolated turtle position (black arrows). Major surface circulation features of the western Indian Ocean during the summer monsoon are shown in blue, including the East African Coastal Current (EACC), the Somali Current (SC), the Great Whirl (GW), and the Summer Monsoon Current (SMC). Insets show key moments of the trajectory overlaid on GEBCO bathymetry: (B) the turtle spent 7 days on a submerged reef, the Banc du Geyser; (C) the turtle turned southward in the open ocean; and (D) during the final segment of the track, the turtle oriented westward toward Huvadhu Atoll.

### 3.2 Ontogenetic Stage

Determining whether this individual had already completed its ontogenetic transition from oceanic to neritic habitat remains challenging. With a CCL of 38 cm, *Naty* falls slightly above the upper size range reported for juveniles transitioning from oceanic to coastal habitats in the western Atlantic (15.7–35.0 cm; Avens et al. 2021). Although transition sizes likely vary among populations and regions, comparable data are currently lacking for the Indian Ocean.

Long‐distance movements between developmental habitats have nevertheless been documented in juvenile hawksbills (Laforge et al. 2026; Mortimer et al. 2010; Whiting et al. 2010; Whiting and Koch 2006). For example, von Brandis et al. (2017) described a 40.3 cm CCL juvenile that departed from a resident developmental habitat in St. Joseph Atoll, Seychelles and traveled approximately 1400 km to the Kenyan coast. The track reported here therefore falls within the range of movements already observed in large juveniles, although its scale is exceptional.

Still, it remains unclear whether this individual was recruiting to a neritic habitat for the first time or relocating between previously established developmental habitats. What is clear, however, is that the two periods of restricted movement observed along the track, the initial 7‐day exploration of the submerged Banc du Geyser immediately after release, and the subsequent 37‐day residency at Huvadhu Atoll indicate that settlement in a neritic habitat was this individual's ultimate objective.

### 3.3 Oceanic Context

Even if the final objective was to settle in a neritic habitat, the individual likely did not initially target the Maldives, a habitat located at a straight‐line distance of 3545 km from the release site in the Mozambique Channel. This is further supported by the turtle's reversal of direction in the open ocean (Figure 1C), north of the Comoros archipelago heading in the opposite direction of the Maldives. During this trajectory shift, the encountered currents were predominantly zonal, pushing westward at 0.4–0.8 km/h and therefore cannot account for the observed southward movement. This suggests that the reversal reflected an active behavioral response, potentially corresponding to a reorientation after overshooting a southward target. The individual might have failed to approach a potential habitat closely enough before being entrained by the powerful Somali Current, the Great Whirl and Summer Monsoon Current potentially taking it far from its initial search area (Figure 1; animation in Supporting Information [Video 1]). Notably, the turtle's journey might have unfolded quite differently had it reached the Kenyan coast several months later, during winter, when the Somali Current flows southward (Schott et al. 2009).

**VIDEO 1:** 12 h interpolated track of Naty overlapping surface currents extracted from GLORYS12. Video content can be viewed at https://onlinelibrary.wiley.com/doi/10.1002/ece3.74475.

Overall, collocation with GLORYS12 revealed that the turtle experienced a mean current speed of 1.6 km/h (SD = 1.4 km/h; range: 0.1–9.2 km/h). Integrating current velocities along the track suggests that approximately 6747 km, or 74% of the total traveled distance, can be attributed to passive transport by oceanic currents. Although this estimate is subject to uncertainties associated with drift estimation (Gaspar et al. 2025), track interpolation, and the assumption of surface drift despite potential diving behavior (Laforge et al. 2023), the magnitude of the estimated advection strongly suggests that regional circulation played a dominant role in shaping the overall trajectory, with a comparatively minor contribution from active swimming.

### 3.4 Active Habitat Selection

While the overall trajectory appears strongly influenced by ocean circulation, the final portion of the track provides evidence of active habitat targeting. Huvadhu Atoll was initially bypassed by approximately 50 km to the east before the turtle abruptly changed direction on 21 October 2010 and swam westward toward the atoll. Surface currents in the wake of the island were weak and could not account for this movement (Figure 2K), indicating active swimming behavior.

**FIGURE 2:**
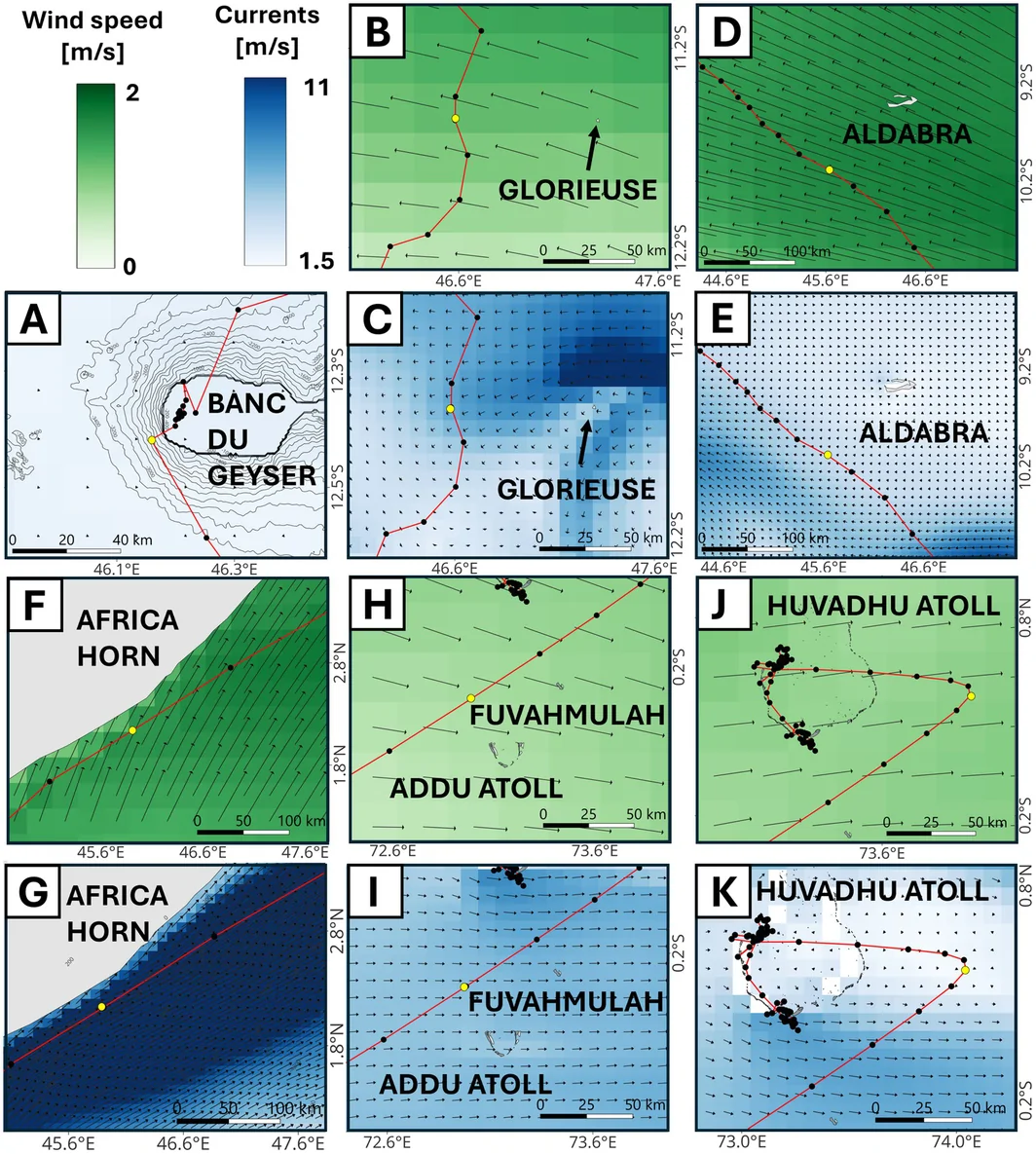
Seven selected segments of the track of the juvenile hawksbill turtle Naty (red line), corresponding to times when the individual passed within 100 km of potential neritic habitats. For each segment, panels show 10 m wind speed from the ERA5 reanalysis (1/4° resolution) on the top panels (B, D, F, H, J) and surface currents from GLORYS12 (1/12° resolution) on the bottom panels (A, C, E, G, I, K). Background shading represents daily wind speed or current speed as appropriate, arrows indicate direction, black points show daily interpolated positions, and the yellow point marks the position corresponding to the day displayed in the background. Note that no wind panel is shown for the Banc du Geyser (A), as wind is not relevant to a submerged reef. The seven segments correspond to (A) the Banc du Geyser, (B, C) Glorieuse Island, (D, E) Aldabra Atoll, (F, G) the Horn of Africa, (H, I) Fuvahmulah and Addu Atoll, and (J, K) Huvadhu Atoll, where the turtle ultimately settled. In all cases, the distance between the track and the nearby landmass was small enough (< 100 km) that airborne or waterborne cues could potentially have been detected by the turtle. Notably, only at the Banc du Geyser (A) and Huvadhu Atoll (J, K) did the turtle show a marked change in trajectory toward the habitat.

Given the very low elevation of coral atolls (approximately 1 m above sea level), visual detection at such distances is unlikely, leaving chemoreception as the most plausible sensory basis for the observed orientation. Sea turtles possess a well‐developed olfactory system and are experimentally known to detect odorants both dissolved in water (Grassman and Owens 1987; Manton et al. 1972) and carried in air (Endres et al. 2009). Crucially, juvenile loggerheads respond to airborne volatiles emanating from coastal mud, a capability interpreted as a mechanism for sensing nearby land (Endres and Lohmann 2013), while tracking and displacement experiments indicate that turtles can locate oceanic islands from distances of several 10 of kilometers (Hays et al. 2003; Luschi et al. 2001). Coral reefs and atolls are, moreover, areas of elevated biological productivity that release dimethyl sulfide (DMS), a volatile compound detected by loggerheads and thought to mark productive waters (Endres and Lohmann 2012); the airborne plume extending downwind of Huvadhu could therefore have carried a distinctive chemical signature of the atoll.

Waterborne cues offer a complementary pathway. Post‐emergent loggerhead hatchlings can already discriminate coastal from oceanic seawater in two choice trials (Soeiro et al. 2022), indicating that chemical detection of land proximity is available early in ontogeny and could plausibly persist in the juvenile stage. Considering the first potential habitat encountered along the track, the Banc du Geyser, the turtle passed only 2.5 km south of the submerged reef before changing course toward it. At this location, the reef was surrounded by waters exceeding 2000 m in depth, with no apparent shallow bathymetric gradient that could have provided a close‐range physical cue to its presence (Figure 1B). Waterborne chemical cues may therefore have provided one possible source of information for detecting the reef. Although current speed was very low (0.65 km/h; Figure 2A), the flow was directed toward the reef, potentially allowing chemical cues to be transported downstream over this short distance. This first course change is therefore consistent with, but does not demonstrate, a possible role for waterborne chemical cues in habitat detection. At the second site where the turtle settled, Huvadhu Atoll, Naty passed approximately 50 km from the atoll, both downstream and downwind of it (Figure 2F,J). Thus, either waterborne or airborne cues, or both, could potentially have conveyed information about the atoll, and the available data do not allow these pathways to be distinguished.

More broadly, the two‐scale pattern documented here, large‐scale displacement dominated by passive advection, followed by localized active orientation once a target became detectable, closely mirrors the proposed two‐step model of sea turtle navigation, in which large‐scale cues bring an individual into the vicinity of a goal while short‐range chemical cues guide the final approach (Endres et al. 2016). Chemosensory imprinting, demonstrated in juvenile green turtles (Grassman and Owens 1987), could additionally refine the recognition of suitable developmental habitat.

To evaluate whether the turtle may have responded similarly to habitats encountered earlier in its journey, we examined all instances where the individual passed within approximately 100 km of land (Figure 2). These included Glorieuses islands (Figure 2B,C), Aldabra Atoll (Figure 2D,E), the Somali coast (Figure 2F,G), and the Addu and Fuvahmulah atolls (Figure 2H,I). Among these locations, only Glorieuse island appears to have been approached from the downwind and downstream side, potentially exposing the turtle to waterborne and airborne chemical cues. At that time, however, the individual was embedded within the strong East African Coastal Current, which transported it westward at velocities approaching 3.6 km/h. Whether the turtle detected the island and attempted to approach it cannot be determined from the available data, but any such response may have been constrained by its limited swimming capacity at that stage and the strong westward flow of the East African Coastal Current, which could have prevented it from reaching the island.

These indirect indications of habitat detection would benefit from additional observations of late‐oceanic‐stage individuals recruiting to neritic habitats while downwind and/or downstream of potential settlement sites, since direct experimental tests of island targeting are highly unlikely to be feasible, given the inherent difficulty of locating and capturing a cohort of turtles immediately prior to this life‐stage transition.

## 4 Implications for Juvenile Habitat Selection

This exceptional track offers one of the few observations of a juvenile hawksbill turtle nearing the end of its oceanic phase, and its final course correction provides, to our knowledge, a first indirect indication that island detection via olfactory cues could guide neritic recruitment in a juvenile sea turtle. This mechanism had only been documented in adult breeding females returning to natal nesting sites (Lohmann and Lohmann 2019), and its extension to the juvenile recruitment stage could suggest that olfactory‐mediated orientation toward suitable habitat is not restricted to reproductive contexts but may be a more general feature of close‐range sea turtle navigation. At the same time, the broader trajectory indicates that dispersal during, or shortly after, the oceanic‐to‐neritic transition remains strongly influenced by regional ocean circulation, even in juveniles approaching 40 cm CCL, with active habitat selection confined to the final stages of settlement once suitable habitat comes within olfactory range. Consequently, the location of juvenile developmental habitats may depend, at least in part, on chance encounters mediated by oceanographic processes, encounters that only translate into directed settlement once the turtle can detect the island itself, with potentially important consequences for subsequent adult migratory pathways.

## Author Contributions

**Antoine Laforge:** conceptualization (equal), formal analysis (lead), investigation (lead), methodology (equal), software (lead), visualization (lead), writing – original draft (lead). **Phillipe Gaspar:** investigation (supporting), writing – review and editing (equal). **Jérôme Bourjea:** data curation (lead), investigation (equal), writing – review and editing (equal). **Manon Nivière:** writing – review and editing (equal). **Katia Ballorain:** funding acquisition (equal), investigation (equal), supervision (lead), writing – review and editing (equal). All authors gave final approval for publication and agreed to be held accountable for the work performed therein.

## Funding

This work was supported by the Global Environment Facility Trust Fund and UE‐INTERREG VI Indian Ocean.

## Conflicts of Interest

The authors declare no conflicts of interest.

## Supporting information


**Data S1:** ece374475‐sup‐0001‐Supinfo1.csv.

**Data S2:** ece374475‐sup‐0002‐Supinfo2.csv.

## Data Availability

The satellite tracking data supporting this study are archived in Zenodo (DOI: https://doi.org/10.5281/zenodo.21371968). Access is currently restricted during peer review and will be made publicly available upon publication.
